# Public policies on sexually transmitted infections in Brazil

**DOI:** 10.1590/0037-8682-611-2020

**Published:** 2021-05-17

**Authors:** Angélica Espinosa Miranda, Francisca Lidiane Sampaio Freitas, Mauro Romero Leal de Passos, Miguel Angel Aragón Lopez, Gerson Fernando Mendes Pereira

**Affiliations:** 1 Ministério da Saúde, Secretaria de Vigilância em Saúde, Brasília, DF, Brasil.; 2 Universidade Federal do Espírito Santo, Vitória, Brasil.; 3 Universidade de Brasília, Programa de Pós-Graduação em Saúde Coletiva, Brasília, DF, Brasil.; 4 Universidade Federal Fluminense, Departamento de Microbiologia e Parasitologia, Niterói, RJ, Brasil.; 5 Organização Pan-Americana da Saúde, Unidade Técnica de Doenças Transmissíveis e Determinantes Ambientais da Saúde, Brasília, DF, Brasil.

## INTRODUCTION

This article presents a brief history of public policies to address sexually transmitted infections (STI) in Brazil. It also discusses deficiencies and challenges to be considered by the country to formulate and implement future policies.

## GLOBAL OVERVIEW, REGION OF THE AMERICAS AND BRAZIL

STI remains a global public health issue. In 2016, the World Health Organization (WHO) estimated a 376.4 million incidence of curable STI in people aged 15 to 49. There were 127.2 million cases of chlamydia, 86.9 million cases of gonorrhea, and 6.3 million cases of syphilis. In the Region of the Americas, an estimated 29.8 million cases of chlamydia, 13.8 million cases of gonorrhea, and 2 million cases of syphilis were reported^1^.

These estimates indicate a high STI incidence and warrant the WHO global strategy and the establishment of priority measures to achieve STI elimination goals by 2030. This strategy focuses on (i) gonococcal antimicrobial resistance and chlamydial co-infection risk, (ii) congenital syphilis elimination, which requires increased testing and treatment of pregnant women and specific populations, and (iii) human papillomavirus (HPV) infection, with a focus on immunization to eliminate cervical cancer and anogenital warts^2^.

The Pan American Health Organization's (PAHO) Plan of Action for the Prevention and Control of HIV/STI (2016-2021) aims at expediting the elimination of the human immunodeficiency virus (HIV) and STI epidemics as public health issues in the Region of the Americas by 2030^3^. This plan incorporates regional strategies for the elimination of HIV and congenital syphilis vertical transmission, with specific criteria and indicators.

In Brazil, acquired syphilis, syphilis in pregnant women, and congenital syphilis cases are compulsorily notifiable^4^. Brazilian epidemiological data highlight the increase in syphilis cases for the period 2010-2018, when the congenital syphilis incidence rate increased almost four times, from 2.4 to 9.0 cases per thousand live births, and the syphilis detection rate in pregnant women increased about six times, from 3.5 to 21.4 cases per thousand live births. The acquired syphilis detection rate increased from 34.1 cases per 100,000 population in 2015 to 75.8 cases per 100,000 population in 2018^5^.

Brazilian nationwide-covering STI prevalence studies reveal the scale of the problem. Parturients attended in public maternity hospitals had a 9.8% prevalence of chlamydia and 1.0% of gonorrhea in 2011^6^, and men who sought care in STI clinics in 2005, 13.1% of chlamydia and 18.4% of gonorrhea^7^. In 2015, women living with HIV had a 2.1% prevalence of chlamydia and 0.9% of gonorrhea^8^, and 28.4% of high-risk HPV^9^. In 2017, the prevalence of HPV infection was 25.4% in the cervix, 36.2% in the penile region, 25.7% in the anal area, and 11.9% in the oral region^10^. Another national study reported a 0.6% prevalence of syphilis in conscripts^11^. In 2016, higher syphilis rates were observed in key population segments, such as men who have sex with men (9.9%)^12^, female sex workers (8.5%)^13^ and prisoners (3.8%)^14^.

## HISTORY OF THE RESPONSE TO IST IN BRAZIL

**FIGURE 1:**
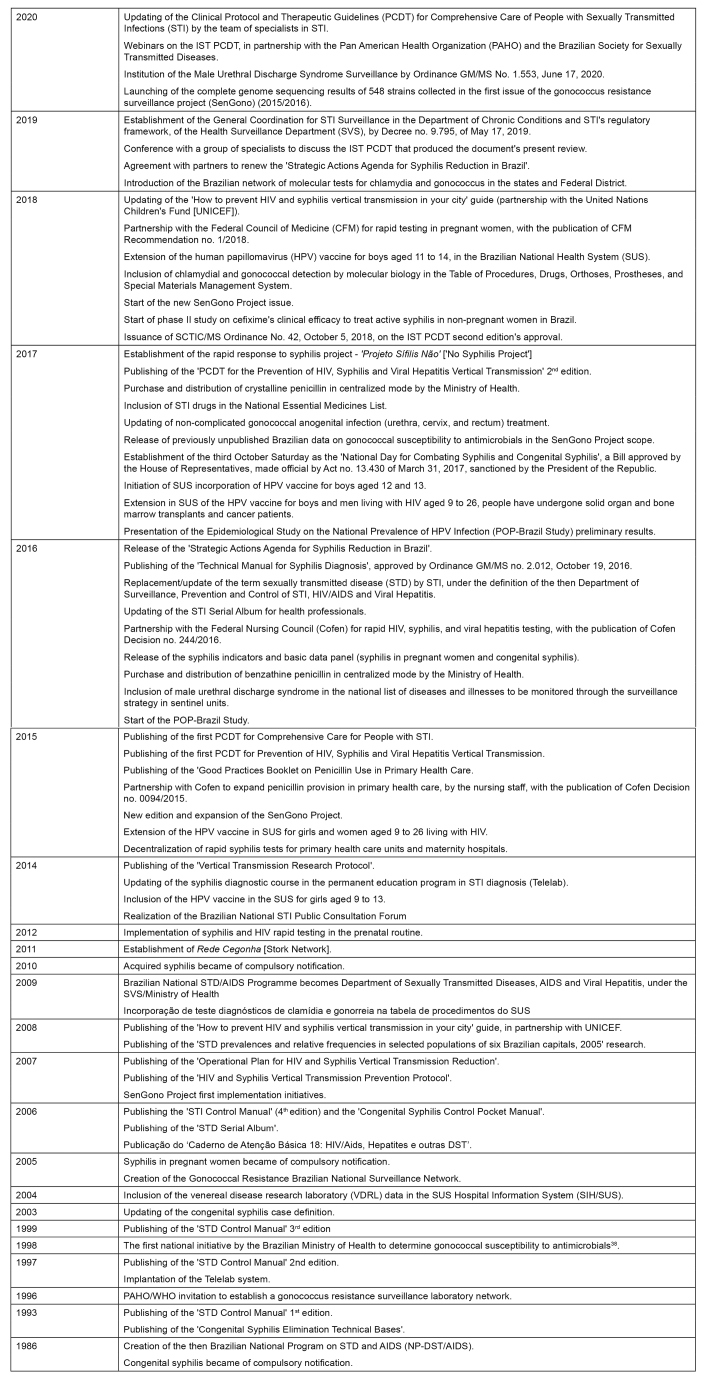
Historical milestones of the response to sexually transmitted infections in Brazil, 1986-2020.

Figure 1 outlines the historical milestones of major strategies and actions within the STI public policy scope in Brazil since creating the Brazilian National Program on Sexually Transmitted Diseases and AIDS (PN-DST/AIDS) in 1986.

The publishing of the first Clinical Protocol and Therapeutic Guidelines (PCDT) for Comprehensive Care for People with STI, approved by the National Committee for the Incorporation of Technologies in the Brazilian National Health System (Conitec), in 2015, is noteworthy^15^. The document establishes criteria for diagnosis, advocates treatment, sets clinical control mechanisms - to be followed by the Brazilian National Health System (SUS) managers - is grounded in scientific evidence, and assesses efficacy, safety, effectiveness, and cost-effectiveness parameters of the recommended technologies^16^. In that publication, the term sexually transmitted disease was replaced by STI, thereby matching the WHO designation^3^ and raising Brazil's awareness of asymptomatic infections and not just illness with signs and symptoms. The PCDT was re-examined and approved by Conitec in 2018^17^.

In the period 2015-2016, the Ministry of Health's partnership with the Federal University of Santa Catarina and sentinel sites for the elaboration of a gonococcal resistance study (SenGono Project) made possible the first Brazilian gonococcal antimicrobial susceptibility surveillance program. The nationwide surveillance research analyzed gonococcal strains at seven sentinel sites (composed of STI services and local support laboratories) and found high tetracycline, penicillin, and ciprofloxacin resistance in the bacteria^18^.

In 2018, a new phase of the SenGono Project started with installing more collection sites and the inclusion of two new antimicrobials (spectinomycin and gentamicin) in the analyses. Within the SenGono Project, the following are undergoing research: *Neisseria gonorrhoeae*, *Chlamydia trachomatis*, *Mycoplasma genitalium,* and *Trichomonas vaginalis*, in urethral discharge samples; and herpes simplex virus types 1 and 2, *Treponema pallidum* and *Haemophilus ducreyi*, in genital ulcer samples^19^ *.*

The SenGono Project results led to the publication, on June 17, 2020, of Ordinance GM/MS no. 1.553/2020, by which the Ministry of Health instituted the sentinel surveillance of male urethral discharge syndrome to monitor data in qualified health units^20^.

Considering the global and national penicillin supply shortage and the difficulties for its acquisition by states and municipalities in the period 2014-2016, the publication of the 'Strategic Actions Agenda for Syphilis Reduction in Brazil' in 2016^21^, as a response to the epidemic declared by the Ministry of Health, played a critical role in the decision for the centralized acquisition of these drugs, reserved to the same Ministry of Health. Benzathine and crystalline penicillin for syphilis treatment became part of the pharmaceutical assistance strategic component in Brazil. Doxycycline was also expanded for the treatment of syphilis, donovanosis, and pelvic inflammatory disease^22^. The penicillin mentioned above global shortage led to the search for effective alternatives for syphilis treatment. For example, in 2018, phase II of a clinical trial was initiated to evaluate the efficacy of cefixime in the treatment of active syphilis in non-pregnant women, establish safe alternative therapeutic options, and support efforts to end congenital syphilis^23^.

Following the HPV vaccination inclusion in SUS, the need for monitoring the impact of such immunization on the specific population was perceived. Research on HPV prevalence in Brazil was scarce until then^10^. In 2016, an HPV infection prevalence national study was started (POP-Brazil Study), a Ministry of Health partnership, amongst other institutions, with the Moinhos de Vento Hospital Association of Porto Alegre, RS, Brazil. The research sought to determine the HPV prevalence in sexually active people, aged 16 to 25, in all Brazilian capitals, and to investigate regional differences in prevalence and viral types; its final results will fill the epidemiological information gap, and contribute to establish a baseline and assess the HPV vaccination impact. The research's preliminary results presented publicly already in 2016, estimated a high HPV prevalence (54.6%), of which 38.4% were high-risk HPV for cancer risk^24^.

## CHALLENGES AND DEFICIENCIES FOR STI CONTROL

The fast antimicrobial resistance growth of *Neisseria gonorrhoeae* to several antibiotics threatens the efforts for controlling this infection. 66% of the 67 countries participating in the Gonococcal Antimicrobial Surveillance Programme have already shown increased clinical and in vitro resistance to broad-spectrum cephalosporins in 2009-2014, and it is the only remaining first-line monotherapy for gonorrhea control^25^. Research developments must have priority, including the development of new antimicrobials for treatment, of a gonococcal vaccine, and new rapid tests, with simultaneous detection of both the gonococcus and antimicrobial resistance, for diagnosis and surveillance^26^.

Some challenging goals in syphilis control include eliminating vertical transmission, improving case surveillance, developing more accurate tests to diagnose active syphilis, neurosyphilis, and congenital syphilis, increasing access for the most vulnerable populations, and developing alternative oral drugs and vaccines against *Treponema pallidum* ^27^.

Lack of knowledge and fake news on immunization are examples of contributing factors to low vaccination coverage for HPV. Health care integration with schools and communities is central to achieve better indicators since well-informed adolescents are potential communicators of such information to their parents^28^ ^,^ ^29^. It is also relevant to promote health information, education, and communication aimed at professionals in the field to expand vaccination coverage^28^ ^,^ ^30^.

## FINAL CONSIDERATIONS

Challenges and deficiencies persist in the formulation and implementation of public policies in IST in Brazil. In such a scenario, there is a permanent need for (i) strengthening the role of primary health care in comprehensive care to people with STI and their sex partners, (ii) ensuring adequate vaccination coverage against HPV and viral hepatitis A and B, (iii) promoting health information, education, and communication, (iv) expand access to STI testing and treatment, with emphasis on the most vulnerable populations, (v) notify sex partners, and (vi) qualify health professionals' approach to sexual health matters, in addition to screening for asymptomatic, prevention, clinical-laboratory management, and surveillance of sexually transmitted infection cases. 
